# Clinical, Immunological, and Environmental Determinants of Pediatric Respiratory Disorders: A Comprehensive Review of Long-Term Health Outcomes

**DOI:** 10.7759/cureus.117121

**Published:** 2026-09-29

**Authors:** Jignesh Sharma, Konda Shravan Kumar, Ankit Sharma, Muzahid K Sheikh, Ashish Kumar Shukla, Saira Sultana Shaikh

**Affiliations:** 1 Department of Pediatrics, Mansarovar Medical College, Sehore, IND; 2 Department of Pediatrics, VV Giri University, Siwan, IND; 3 Department of Pediatrics, Subharti Medical College, Swami Vivekanand Subharti University, Meerut, IND; 4 Department of Neuro Physiotherapy, Datta Meghe College of Physiotherapy, Nagpur, IND; 5 Department of Radiodiagnosis, Santosh Medical College, Santosh Deemed to be University, Ghaziabad, IND; 6 Department of Botany, P.K. University, Shivpuri, IND

**Keywords:** air pollution, asthma, immunology, pediatric respiratory disorders, wheezing

## Abstract

Pediatric respiratory disorders remain a major source of childhood morbidity, healthcare utilization, impaired quality of life, and long-term pulmonary vulnerability. The background of this review centers on the complex interaction between early-life clinical susceptibility, immune maturation, environmental exposure, respiratory infections, and social determinants in shaping pediatric airway health. The objective was to synthesize evidence on clinical, immunological, infectious, environmental, and social determinants relevant to pediatric asthma, recurrent wheeze, and associated long-term respiratory outcomes, including disease persistence, impaired lung-function trajectories, and later respiratory vulnerability. The methodology involved a structured literature search of studies published from 2015 to 2025 in PubMed, Scopus, Web of Science, Embase, and Google Scholar, including observational studies, clinical studies, systematic reviews, meta-analyses, and high-quality narrative reviews focused on infants, children, and adolescents. The evidence indicates that early-life respiratory vulnerability, recurrent viral infections, atopic and inflammatory pathways, microbiome alterations, environmental exposures, and social disadvantage may interact across childhood and contribute to heterogeneous long-term respiratory trajectories. Longitudinal cohort findings further show that some children maintain persistent airflow impairment into adulthood, whereas others demonstrate partial improvement over time. Because the available evidence is stronger for associations and prognostic trajectories than for intervention efficacy, preventive and clinical strategies should be interpreted cautiously, with emphasis on appropriate diagnosis, phenotype- and severity-based management, reduction of avoidable exposures, age-appropriate pulmonary-function monitoring, and equitable access to follow-up.

## Introduction and background

Pediatric respiratory diseases are a major clinical and public health problem, contributing to childhood morbidity, healthcare utilization, impaired quality of life, and potentially adverse respiratory outcomes later in life [1]. Chronic respiratory morbidity originating in childhood may also contribute to respiratory impairment extending into later life [2]. Childhood is a critical period of pulmonary development during which disruption of airway and lung growth can influence subsequent respiratory function [3]. Environmental exposures occurring during these developmental periods may further modify respiratory susceptibility and long-term airway health [4]. Environmental contributions to pediatric respiratory disease encompass both prenatal and postnatal exposures that can interact with the developing respiratory system [5]. Although pediatric respiratory morbidity encompasses a broad range of conditions, the present review primarily focuses on asthma and recurrent wheeze as long-term respiratory outcomes. Bronchiolitis is considered an important early-life respiratory illness because of its substantial childhood disease burden and relevance to subsequent respiratory morbidity [6].

Among these conditions, asthma and recurrent wheeze are particularly relevant because of their high prevalence, recurrent symptom burden, medication requirements, and potential for exacerbations [1]. Socioeconomic disadvantage may further modify pediatric respiratory risk through clustering of adverse environmental and living conditions [7]. Household tobacco smoke and e-cigarette aerosol are additional potentially harmful inhalational exposures; epidemiological and laboratory evidence has linked e-cigarette exposure with adverse respiratory effects [8].

Social and environmental determinants may operate from early life and influence respiratory health through housing conditions, pollutant exposure, healthcare access, and other structural factors [9]. Immune and developmental mechanisms also provide important biological links between early-life exposures and persistent respiratory disease. Developmental immunity and early respiratory infections can interact to influence lifelong lung health, including subsequent wheeze and asthma susceptibility [10]. Rather than attributing long-term outcomes to a single immunological pathway, this review considers how host susceptibility, respiratory infections, inflammatory responses, microbiome alterations, and environmental exposures may interact during childhood.

Environmental exposure is another important determinant of pediatric respiratory health. The pediatric exposome includes multiple interacting outdoor, indoor, behavioral, and social exposures that may act across different developmental periods [4]. Climate-related factors, including temperature variation, altered aeroallergen exposure, pollution patterns, and extreme environmental events, have also been associated with childhood and adolescent asthma [11]. These exposures may coexist rather than act independently, complicating estimation of the contribution of individual determinants.

These determinants should therefore not be viewed as isolated domains. A conceptual framework for this review considers early-life developmental susceptibility as the underlying context within which respiratory infections, environmental exposures, immune responses, and social conditions interact over time. Repeated or cumulative insults may influence whether respiratory symptoms resolve, persist as asthma or recurrent wheeze, or contribute to impaired lung-function development and later respiratory vulnerability. Longitudinal birth-cohort evidence further demonstrates that childhood lung-function patterns can track into adulthood and that early-life characteristics are associated with persistently lower respiratory trajectories [12].

Although substantial evidence has examined childhood asthma, environmental exposures, early-life respiratory risk, and chronic respiratory morbidity, much of this literature addresses individual diseases or determinants separately [1,4,5,9-12]. Therefore, the gap addressed by the present review is not an absence of previous evidence, but the limited integrative synthesis of interacting clinical, immunological, infectious, environmental, and social determinants across childhood and their relationship with long-term respiratory trajectories. This review differs from narrower disease-specific or exposure-specific literature by considering these domains within a common developmental framework. The resulting synthesis is intended to identify areas relevant to risk stratification and longitudinal surveillance while distinguishing established associations from areas in which causal or intervention evidence remains limited.

Objectives of the review

This review aims to synthesize current knowledge on clinical, immunological, environmental, infectious, and social determinants relevant to pediatric asthma, recurrent wheeze, and associated long-term respiratory outcomes. It examines how these determinants interact with respiratory development and evaluates their relationships with disease persistence, lung-function trajectories, and later respiratory vulnerability, while identifying limitations in current evidence and priorities for future research.

Methodology

Search Strategy

A structured literature search was conducted to identify relevant studies addressing clinical, immunological, infectious, environmental, and social determinants of pediatric respiratory disorders and associated long-term respiratory outcomes. The final database search was conducted on 8 December 2026 and was restricted to publications dated from January 2015 through December 2025. PubMed, Scopus, Web of Science, Embase, and Google Scholar were searched using combinations of controlled vocabulary and free-text terms related to pediatric respiratory disease, asthma, recurrent wheeze, bronchiolitis, pneumonia, bronchopulmonary dysplasia, airway inflammation, immune development, respiratory syncytial virus, rhinovirus, microbiome, air pollution, indoor allergens, tobacco smoke exposure, climate-related exposure, social determinants, lung-function trajectories, and long-term respiratory outcomes. Boolean operators (“AND” and “OR”) were used to combine disease-, determinant-, exposure-, and outcome-related concepts. A representative search structure combined respiratory-condition terms with determinant/exposure terms and long-term outcome terms, and the syntax was adapted to the indexing requirements of each database. Reference lists of relevant review articles and selected primary studies were additionally screened to identify potentially relevant publications. Because this was designed as a structured narrative review rather than a systematic review, the search aimed to identify clinically relevant evidence across predefined domains and did not claim exhaustive retrieval of all eligible publications. Accordingly, formal PRISMA reporting and a PRISMA study-selection flow diagram were not applied.

Eligibility and Study Selection

Eligible publications included original observational studies, cohort studies, case-control studies, cross-sectional studies, clinical studies, systematic reviews, meta-analyses, and high-quality narrative reviews published in English between 2015 and 2025. Studies involving infants, children, or adolescents were eligible when they addressed asthma, recurrent wheeze, or related long-term respiratory outcomes. Studies evaluating bronchiolitis, pneumonia, or bronchopulmonary dysplasia were included when they provided evidence concerning early-life respiratory vulnerability, subsequent wheezing or asthma risk, impaired lung-function development, or other relevant long-term outcomes. Studies addressing clinical, immunological, infectious, environmental, social, or longitudinal determinants of pediatric respiratory health were also considered eligible. Adult-only studies, non-English publications, editorials, letters without primary or synthesized evidence, conference abstracts without full text, animal-only studies, publications outside the prespecified period, and studies lacking relevance to pediatric respiratory determinants or long-term health outcomes were excluded.

Titles and abstracts were assessed for relevance to the predefined eligibility criteria, followed by full-text evaluation of potentially eligible publications. Duplicate records, clearly irrelevant publications, and articles that did not satisfy the population, determinant/exposure, outcome, publication period, language, or article-type criteria were excluded. The final narrative synthesis comprised 50 publications. Because this structured narrative review was not prospectively conducted using a systematic review screening protocol, record-level counts for the initial search yield, duplicate removal, title/abstract exclusions, and full-text exclusions were not retained and therefore are not reported.

Methodological Appraisal and Risk of Bias

Because the review incorporated heterogeneous evidence types and was not designed as a systematic review, a single formal risk-of-bias instrument was not applied uniformly across all included publications. Instead, methodological quality and potential sources of bias were considered qualitatively during evidence synthesis. Particular attention was given to study design, participant selection, sample size, exposure and outcome ascertainment, adjustment for potential confounding, completeness and duration of follow-up, and consistency between reported results and study conclusions. For systematic reviews and meta-analyses, consideration was given to the transparency of the search strategy, eligibility criteria, heterogeneity, and limitations reported by the original investigators. Evidence derived primarily from cross-sectional or observational studies was interpreted as associative rather than causal, whereas longitudinal evidence was given greater interpretive weight when assessing developmental and long-term respiratory trajectories. This appraisal was used to contextualize the strengths and limitations of individual findings rather than to assign numerical quality scores or categorical risk-of-bias ratings.

Data Extraction and Narrative Synthesis

Study selection and extraction of relevant information were undertaken by the authors according to the predefined eligibility criteria and thematic domains. Because this was a structured narrative review, independent duplicate screening and formal adjudication procedures were not prospectively specified. Relevant information was extracted according to predefined thematic domains comprising clinical determinants, immunological mechanisms, respiratory infections and microbiome alterations, environmental exposures, social determinants, and long-term respiratory outcomes. Extracted information included study population, study design, determinant or exposure evaluated, principal respiratory outcome, and clinically relevant long-term findings where available.

Evidence was grouped thematically and synthesized narratively. Quantitative pooling was not undertaken because of substantial clinical and methodological heterogeneity in study populations, study designs, exposure definitions, outcome measures, and follow-up periods. Consequently, pooled effect estimates, review-level P-values, 95% confidence intervals, and meta-regression models were not calculated. The numerical estimates presented in the review were derived from individual source studies and reported descriptively rather than recalculated or statistically combined. Greater emphasis was placed on consistency across studies, longitudinal evidence, clinically meaningful outcomes, and areas of uncertainty or conflicting evidence. The synthesis was undertaken to characterize reported determinants and their relationships with persistent wheeze, asthma persistence, impaired lung-function trajectories, and later respiratory vulnerability rather than to establish a quantitative hierarchy of risk. Clinically relevant evidence gaps were defined as areas with limited, inconsistent, or predominantly observational evidence concerning early risk prediction, phenotype-based stratification, integration of multiple determinants, longitudinal follow-up, and effectiveness of preventive interventions.

Statistical Analysis

No formal meta-analysis, meta-regression, pooled effect estimation, or other inferential statistical synthesis was performed. The included literature was heterogeneous with respect to study design, participant characteristics, determinants and exposures, outcome definitions, follow-up periods, and statistical reporting, which precluded clinically meaningful quantitative pooling. Accordingly, no review-level P-values, pooled effect estimates, or 95% confidence intervals were calculated. Numerical estimates reported in the Review section, including proportions and lung-function trajectory data, were reproduced from the individual source studies for descriptive comparison and were not recalculated or statistically combined. Evidence was therefore synthesized narratively, with emphasis on the consistency and direction of reported associations, longitudinal findings, clinical relevance, and areas of uncertainty.

## Review

Epidemiology and clinical burden of pediatric respiratory disorders

Pediatric respiratory disease is a significant contributor to morbidity throughout infancy, childhood, and adolescence and leads to substantial demands for acute care, healthcare utilization, and long-term respiratory morbidity [13]. Within the scope of this review, the principal clinical outcomes are asthma and recurrent wheeze, while bronchiolitis, pneumonia, bronchopulmonary dysplasia, allergic airway disease, and environmentally related respiratory exacerbations are considered when they contribute to subsequent wheezing, impaired lung-function development, or later respiratory vulnerability. For consistency, respiratory disorders in this review are classified according to their predominant clinical role rather than as mutually exclusive categories. Asthma and recurrent wheeze represent the principal long-term airway outcomes; bronchiolitis and pneumonia are considered acute lower respiratory tract illnesses; bronchopulmonary dysplasia is considered a developmental chronic lung disorder; and allergic or environmentally related respiratory manifestations are discussed as modifying factors where relevant. Because overlap among these categories is common in pediatric practice, individual conditions are discussed according to their predominant role within the framework of this review. Asthma is among the most common chronic respiratory disorders of childhood and is characterized by recurrent respiratory symptoms, variable airflow limitation, and episodic exacerbations [1]. Acute lower respiratory tract infections, particularly bronchiolitis and pneumonia, are important causes of childhood morbidity and hospital admission, with bronchiolitis producing a substantial inpatient burden among infants and other vulnerable pediatric groups [6,13].

The clinical burden of pediatric respiratory disease extends beyond disease frequency and includes substantial effects on daily functioning, healthcare use, and quality of life [13]. Severe or recurrent respiratory illness may contribute to repeated emergency visits, hospitalization, treatment requirements, and disruption of normal childhood activities [13,14]. The broader burden of chronic respiratory disease also includes substantial healthcare utilization and longer-term morbidity, although the magnitude varies according to the underlying disorder and disease severity [13,15].

Pediatric respiratory morbidity is highly geographically and socioeconomically variable. Children experiencing socioeconomic disadvantage may have greater exposure to poor housing conditions, indoor and outdoor pollutants, overcrowding, and other adverse environmental conditions associated with respiratory morbidity [7]. These environmental and social risks frequently cluster, making it difficult to isolate the independent contribution of any single exposure [4,7]. Differences in healthcare access and socioeconomic circumstances may also influence disease recognition, continuity of care, and subsequent respiratory outcomes [9]. Understanding epidemiology and the burden of illness is important for identifying vulnerable populations and interpreting the clinical significance of respiratory determinants. Overall, the burden of pediatric respiratory disease is heterogeneous and reflects interactions among disease severity, developmental vulnerability, environmental exposure, and access to care rather than a uniform pattern across all children. Table 1 summarizes the clinical and exposure-related burden of common pediatric respiratory disorders, highlighting major presentations, vulnerable groups, and long-term respiratory consequences.

**Table 1:** Clinical burden and long-term relevance of major pediatric respiratory conditions and exposure groups. Created by the authors based on information synthesized from previously published studies [2,6,13,15]. Therefore, no copyright permission or license agreement was required. ED: emergency department, QoL: quality of life, SHS: secondhand smoke, PM: particulate matter, LRTI: lower respiratory tract infection, ICU: intensive care unit, CHD: congenital heart disease, BPD: bronchopulmonary dysplasia.

Condition or exposure group	Common clinical presentation	Major burden indicators	High-risk pediatric groups	Long-term outcome relevance	References
Asthma and recurrent wheeze	Episodic cough, wheeze, dyspnea, chest tightness	ED visits, controller medication use, school absence, QoL reduction	Children with atopy, early-life respiratory vulnerability, or relevant environmental exposures	Persistent asthma, reduced lung-function trajectory	[6]
Bronchiolitis and viral LRTI	Tachypnea, wheeze, hypoxia, and feeding difficulty	Hospital admission, oxygen therapy, ICU care	Infants, preterm neonates, CHD, BPD	Recurrent wheeze and subsequent airway hyperresponsiveness in susceptible children	[13]
Pneumonia	Fever, cough, respiratory distress, hypoxemia	Antibiotic use, imaging, admission, and mortality risk	Malnutrition, immunodeficiency, and poor vaccine access	Potential for prolonged respiratory morbidity following severe infection	[15]
Environmental exposure-related respiratory morbidity	Cough, wheeze, asthma exacerbations, or allergic respiratory symptoms associated with adverse environmental exposures	Repeated healthcare use and greater symptom burden	Children exposed to air pollution, dampness, mould, overcrowding, or other adverse housing conditions	Persistent respiratory symptoms and potentially impaired lung-function development	[2]

Clinical determinants: Early-life risk factors, comorbidities, and disease phenotypes

Prenatal, perinatal, and early postnatal events are critical periods for airway growth, immune regulation, and pulmonary development, and disruption during these stages may influence respiratory health across the life course [16]. Prematurity and impaired early lung development may predispose children to recurrent wheeze, reduced lung-function growth, and persistent airflow limitation because alveolarization and airway development may remain incomplete at birth [16,17]. Bronchopulmonary dysplasia and other disorders of fetal or neonatal lung development may further contribute to persistent structural and functional pulmonary abnormalities that extend beyond infancy [17]. These early insults can contribute to failure to achieve optimal peak lung function and greater susceptibility to chronic respiratory morbidity later in life [16-18].

Early respiratory morbidity may interact with atopic predisposition, recurrent infections, environmental exposures, and differences in airway-to-lung growth to influence subsequent wheezing and asthma phenotypes [9,16,18]. Persistent wheezing is more likely to occur in children with continuing respiratory vulnerability, atopic features, or impaired lung function, whereas some early-childhood wheezing patterns may diminish as the airways grow [9,18]. Perinatal nutritional and metabolic influences may also contribute to early respiratory development and later chronic lung vulnerability [19].

Daytime sleepiness, reduced exercise capacity, neurocognitive impairment, and altered perception of respiratory symptoms may occur as consequences of sleep-disordered breathing rather than as exacerbations of the disorder [20]. Recognition of distinct respiratory phenotypes is clinically important because transient wheeze, persistent wheeze, and asthma-prone phenotypes may follow different developmental trajectories [9,18]. Some early-childhood wheezing phenotypes may improve or resolve as airway caliber increases with growth, whereas persistent wheezing and asthma-like phenotypes are more commonly associated with ongoing respiratory susceptibility and reduced lung function [9,18].

These clinical patterns also have important long-term implications. Children exposed to prematurity, impaired early lung development, persistent airway inflammation, or recurrent respiratory morbidity may fail to attain optimal peak lung function, resulting in lower respiratory reserve during adolescence and adulthood [16-19]. Persistent airflow limitation or abnormal airway-to-lung growth relationships established during childhood may continue across the life course and increase susceptibility to chronic obstructive respiratory phenotypes in adulthood [20]. Conversely, children with transient wheezing phenotypes and preserved lung growth may experience substantial symptom improvement as the respiratory system matures [18]. Developmental immunity and early respiratory infections may further influence whether these trajectories persist by modifying antiviral responses, inflammatory programming, and subsequent airway susceptibility [21]. Accordingly, the principal value of phenotype recognition in this review is prognostic: it may help identify children who require longer-term clinical assessment and pulmonary-function surveillance rather than implying that any single phenotype determines a fixed outcome. Overall, the available evidence supports an association between early developmental vulnerability and adverse respiratory trajectories, although outcomes remain heterogeneous and cannot be predicted reliably from any single early-life determinant. Prospective studies incorporating repeated pulmonary-function assessment and objective characterization of relevant exposures are needed to improve individual risk stratification.

Immunological development and airway inflammation in childhood respiratory disease

Immunological maturation is a key factor in respiratory morbidity in children, especially infants and young children, when mucosal and systemic immune responses are still developing [21]. Epithelial barrier function, innate antiviral defense, and adaptive immune regulation continue to mature during early childhood, and disturbances in this process may influence susceptibility to infection, allergic sensitization, and persistent airway inflammation [21]. The balance between T-helper 1 and T-helper 2 (Th2) immune pathways has major relevance in childhood airway disease. A Th2 predominance favors signaling by cytokines such as interleukin-4, interleukin-5, and interleukin-13, immunoglobulin E class switching, eosinophil recruitment, mucus hypersecretion, and airway hyperresponsiveness [21,22].

There is a strong association between this type 2 inflammatory profile and atopic asthma, persistent wheeze, allergic rhinitis, eczema, and recurrent exacerbation risk [22]. Persistent inflammatory signaling can contribute to epithelial injury, airway remodeling, smooth muscle reactivity, and progressive airflow limitation [22]. Inflammatory phenotype assessment may incorporate markers such as immunoglobulin E, allergen sensitization, blood eosinophilia, and fractional exhaled nitric oxide, although interpretation depends on age and clinical context [1,22]. Pediatric respiratory vulnerability is also influenced by epithelial barrier dysfunction. Airway epithelium functions as both a physical and immunological barrier and participates in antimicrobial, interferon, chemokine, and cytokine responses to pathogens and inhaled irritants [22]. Environmental exposures may modify epithelial and immune responses relevant to airway inflammation; detailed exposure-specific effects are considered in the Environmental Determinants section [23].

Innate immunity also contributes to early respiratory outcomes through pattern-recognition receptors, macrophages, dendritic cells, neutrophils, natural killer cells, and interferon-mediated antiviral defense [21,22]. Impaired or delayed antiviral responses may increase susceptibility to severe viral lower respiratory tract infection and recurrent wheezing following respiratory syncytial virus (RSV) or rhinovirus exposure [21]. Respiratory viral infections may also interact with the developing airway microbiome, modifying epithelial responses, inflammatory signaling, and subsequent susceptibility to recurrent wheeze [24]. Viral infection itself can alter microbial community structure and facilitate secondary bacterial interactions, demonstrating that infection-related immune effects cannot be considered independently of the airway microbiome [25].

Evidence links severe early-life viral respiratory infections to recurrent wheeze, altered airway reactivity, and later respiratory morbidity [26], supporting the long-term relevance of these interactions. Early immune events may therefore have consequences extending beyond the acute illness period. Repeated infections and antigenic exposures can influence developmental immune programming, while persistent type 2 inflammation and recurrent epithelial injury may contribute to airway remodeling and sustained airflow limitation [21,22,26]. These mechanisms provide a plausible biological link between childhood inflammatory phenotypes and respiratory morbidity extending into adolescence and adulthood.

Evidence that early intervention can permanently redirect immune programming remains limited. Reduction of persistent airway inflammation, harmful environmental exposures, and recurrent respiratory insults may decrease inflammatory burden, but available evidence does not establish that these approaches permanently modify lifelong immune or lung-function trajectories [21-23,26]. Longitudinal studies linking childhood immune phenotypes, repeated biomarker assessment, and adult respiratory outcomes are therefore still needed. Collectively, the evidence supports type 2 inflammation, epithelial dysfunction, and altered antiviral responses as biologically relevant pathways in persistent pediatric airway disease; however, their relative contribution to long-term respiratory trajectories and the extent to which early modification of these pathways alters later outcomes remain uncertain. Figure 1 shows the interaction between immune maturation, environmental and infectious exposures, inflammatory pathways, and resultant clinical outcomes involved in the pathogenesis of childhood wheeze and asthma.

**Figure 1:**
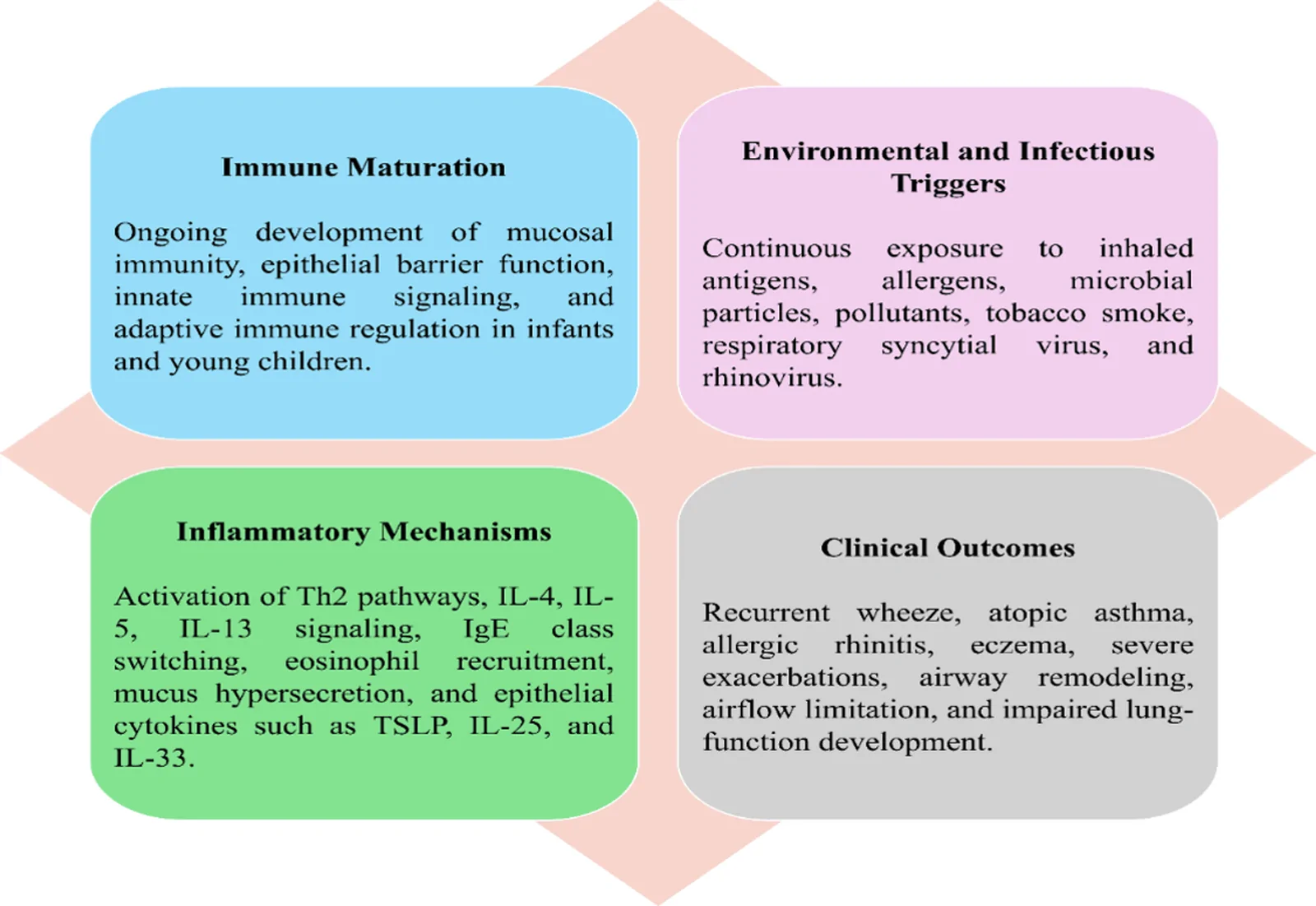
Mechanisms of early childhood wheeze and asthma development. Authors’ own illustration, created using Microsoft PowerPoint based on information synthesized from previously published studies [23-26]. The figure was independently created by the authors and was not reproduced or adapted from any external source. Th2: T-helper type 2; IL: interleukin; IgE: immunoglobulin E; TSLP: thymic stromal lymphopoietin.

Respiratory viral infections, microbiome alterations, and long-term airway outcomes

Early childhood respiratory viral infections play an important role in programming the airway, immune system, and future respiratory morbidity [24]. Rhinovirus and respiratory syncytial virus (RSV) are strongly associated with acute bronchiolitis, recurrent wheezing, airway hyperresponsiveness, and later susceptibility to asthma, particularly in children with underlying atopy, impaired antiviral responses, prematurity, or other clinical susceptibility [24,25]. Severe viral lower respiratory tract infections may contribute to persistent abnormalities in airway reactivity and lung-function development through epithelial injury, inflammatory-cell recruitment, mucus obstruction, and altered airway repair [26]. In infants, RSV bronchiolitis is particularly significant because severe disease occurs during a period of small airway caliber and ongoing immune and epithelial maturation [26]. Severe or recurrent RSV-associated lower respiratory illness may therefore contribute to persistent wheezing and altered airway reactivity beyond the acute infection, particularly in children with pre-existing clinical or immunological susceptibility [24,26].

Rhinovirus-associated wheezing is often associated with allergic sensitization and type 2 inflammation, indicating an interaction between viral infection and atopic immune responses [24]. Rhinovirus infection may amplify epithelial cytokine signaling and type 2 inflammatory responses, particularly in children with established allergic sensitization, thereby contributing to recurrent wheezing and persistence of asthma-prone phenotypes [24]. Viral effects are also influenced by the composition of the airway microbiota. Bacterial genera such as Haemophilus and Moraxella have been associated with pro-inflammatory airway responses and may interact with RSV or rhinovirus to modify the severity of wheezing and airway inflammation [24]. Human infant studies have further shown that early nasopharyngeal microbial composition is associated with the severity of lower respiratory tract infections and subsequent risk of asthma development, supporting a relationship between early microbial colonization and later respiratory outcomes [24,27].

The gut microbiome may influence respiratory immunity through the gut-lung axis. Microbial fermentation products, particularly the short-chain fatty acids acetate, propionate, and butyrate, can participate in immune regulation and may influence systemic and airway inflammatory responses [24]. Early-life disturbances such as antibiotic exposure, cesarean delivery, formula feeding, and recurrent infection can alter microbial diversity and succession, potentially affecting mucosal tolerance and immune maturation [24,27]. Antibiotic exposure is therefore relevant not only because it changes microbial composition but also because it may modify microbial metabolite production and host-microbe signaling during critical periods of immune development [24].

The relationship is bidirectional: respiratory viral infections can alter bacterial community structure, while pre-existing microbial composition may influence susceptibility to infection, inflammatory responses, and subsequent wheezing [24,25]. The available evidence therefore suggests that long-term respiratory outcomes are shaped not by viral exposure alone, but by interactions among viral severity, microbial composition, host immunity, atopic predisposition, and environmental exposures [24-27]. However, considerable uncertainty remains regarding which microbial signatures are causally involved and whether microbiome-directed interventions can modify subsequent asthma or lung-function trajectories. This uncertainty reflects the predominantly associative nature of current human microbiome evidence and the variability in sampling sites, microbial profiling methods, age at assessment, and clinical outcomes across studies [24,27]. Table 2 shows major viral and microbiome-related mechanisms associated with long-term pediatric airway outcomes.

**Table 2:** Viral and microbiome-related determinants of long-term pediatric respiratory outcomes. Created by the authors based on information synthesized from previously published studies [11,23,26-29]. Therefore, no copyright permission or license agreement was required. RSV: respiratory syncytial virus, RV: rhinovirus.

Determinant	Principal pathway	Common clinical association	Long-term airway relevance	References
RSV bronchiolitis	Epithelial injury, mucus plugging, innate immune activation	Infant wheeze, hypoxia, hospitalization	Recurrent wheeze, asthma susceptibility, altered airway reactivity	[28]
RV infection	Impaired antiviral response and interaction with type 2 inflammation	Preschool wheeze, wheeze associated with allergic sensitization	Persistent asthma phenotype, recurrent wheeze and altered airway reactivity	[26]
Microbiome dysbiosis	Reduced microbial diversity, altered immune tolerance	Recurrent infection, allergic sensitization	Airway hyperresponsiveness, asthma persistence, and immune imbalance	[26,29]
Early antibiotic exposure	Gut-airway axis disruption, metabolite alteration	Dysbiosis, recurrent wheeze association	Altered microbial signaling and potential modification of allergic airway risk	[26,29]

Environmental determinants: Outdoor pollution, indoor exposures, and climate-related risks

Environmental exposures are a significant influence on pediatric respiratory morbidity, especially during periods of rapid airway growth and immune maturation [28]. Outdoor pollutants such as particulate matter, nitrogen dioxide, ozone, sulfur dioxide, and traffic-related emissions may promote oxidative stress, epithelial injury, airway inflammation, and impaired mucociliary clearance [29]. Children exposed to higher pollutant concentrations have been reported to experience more frequent cough, wheeze, asthma exacerbations, reduced exercise tolerance, and greater healthcare utilization [30]. Repeated exposure during critical periods of lung development may also contribute to reduced lung-function growth and persistent airway hyperresponsiveness, although the magnitude of effect varies according to pollutant type, exposure intensity, timing, and underlying susceptibility [29-31].

Secondhand tobacco smoke remains an important indoor respiratory irritant associated with recurrent wheeze, poorer asthma control, and lower respiratory morbidity [29]. Other indoor exposures, including biomass combustion, kerosene use, inadequate ventilation, and cooking-related pollutants, may increase particulate and gaseous irritant exposure and contribute to chronic respiratory symptoms [29]. These indoor exposures may coexist and contribute cumulatively to respiratory risk, although their independent effects are difficult to isolate in observational studies [28,29].

Indoor allergens and dampness require separate consideration because their effects involve both allergenic sensitization and non-allergic inflammatory pathways. Exposure to dust mites, cockroach allergens, rodent allergens, pet dander, mould, and damp indoor environments has been associated with wheezing, asthma exacerbations, allergic symptoms, and airway inflammation [31,32]. However, the studies included in this review did not uniformly confirm allergen sensitization using skin-prick testing. Some studies assessed environmental exposure through housing characteristics, questionnaires, environmental measurements, or previously established allergic sensitization rather than direct skin-prick testing. Therefore, these findings should be interpreted as associations between indoor exposures and respiratory outcomes and not as proof that a specific allergen was the direct cause of symptoms in every child.

Environmental burden may also vary geographically and socially, because residential proximity to traffic or industrial sources and neighborhood-level disadvantage can influence the intensity and clustering of respiratory exposures [28,33]. This heterogeneity is important when interpreting associations between environmental determinants and pediatric respiratory outcomes. Climate-related exposures may influence pediatric respiratory disease through several mechanisms rather than through temperature change alone. Rising temperatures and longer pollen seasons can increase the duration and intensity of aeroallergen exposure, whereas heat and atmospheric conditions can alter ozone formation and pollutant concentrations. Wildfire smoke and drought-related dust increase exposure to fine particulate matter and airway irritants, while flooding and persistent dampness can promote indoor mould growth.

These pathways may increase epithelial injury, oxidative stress, allergic inflammation, and asthma exacerbations, particularly in children with pre-existing atopy or chronic airway disease [11]. Current evidence also suggests that climate-related respiratory effects vary according to exposure timing, duration, geographic setting, and underlying susceptibility rather than producing a uniform effect across children [11]. The long-term effect is likely to depend on the timing, duration, and recurrence of exposure during periods of lung growth, with repeated environmental insults potentially contributing to persistent symptoms and suboptimal lung-function trajectories [11,29-31]. Overall, the available evidence consistently associates adverse environmental exposures with pediatric respiratory morbidity, but substantial heterogeneity in exposure assessment, timing, intensity, co-exposures, and residual confounding limits causal attribution and prevents direct comparison of the relative effects of individual environmental determinants. Evidence that modification of a single exposure produces sustained improvement in long-term lung-function trajectories also remains limited. Figure 2 shows the principal pathways linking outdoor pollution, indoor irritants and allergens, and climate-related exposures with pediatric respiratory morbidity and long-term airway outcomes.

**Figure 2:**
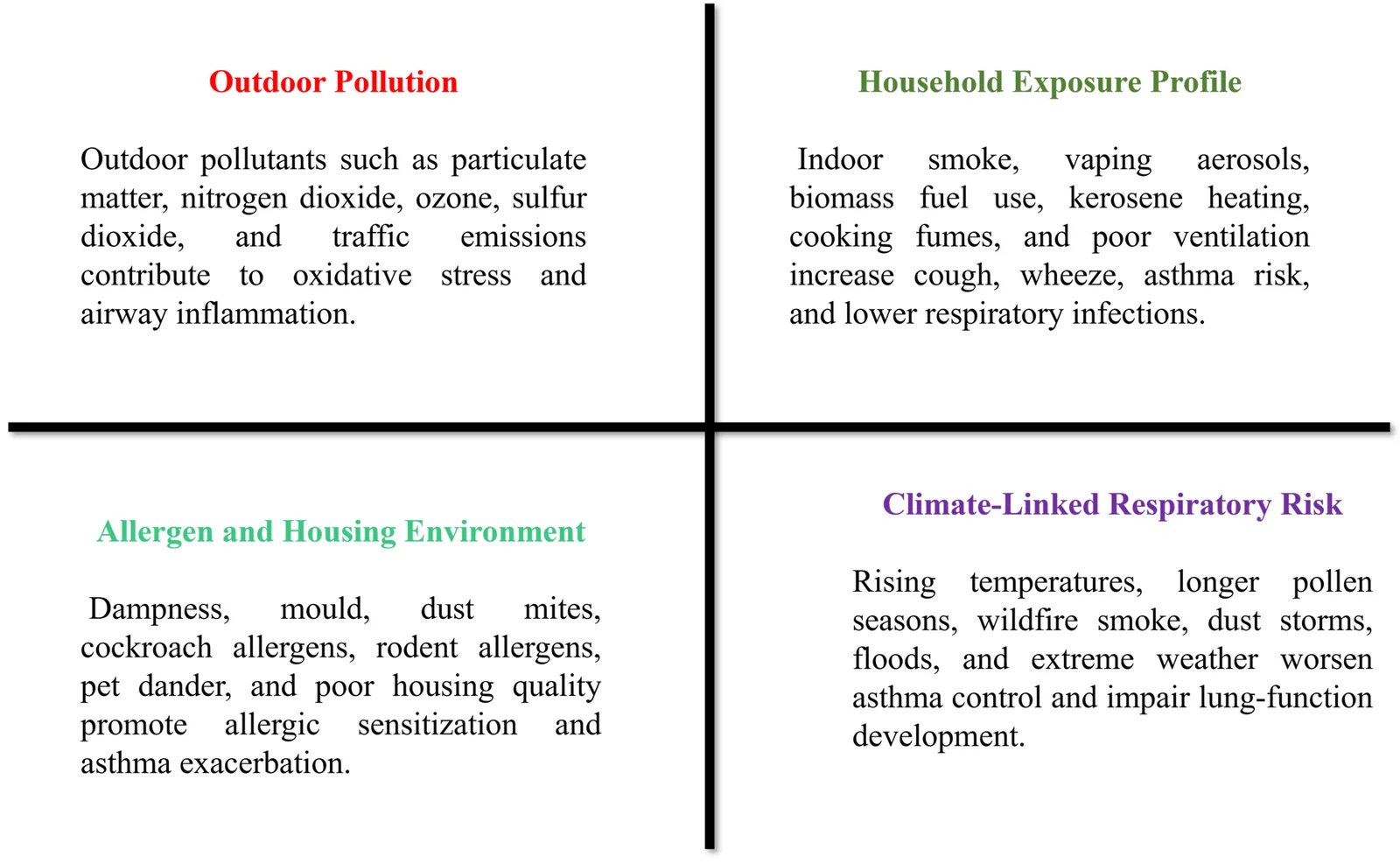
Environmental and climate-related risk factors for childhood respiratory disease. Authors' own illustration, created using Microsoft PowerPoint based on information synthesized from previously published studies [1-3,10,25,31-34]. The figure was independently created by the authors and was not reproduced or adapted from any external source.

Social determinants, health inequities, and access to pediatric respiratory care

Social determinants play a significant role in pediatric respiratory morbidity by influencing exposure patterns, disease recognition, access to healthcare, continuity of care, and clinical outcomes [9,28]. In the studies reviewed, “social determinants” were not represented by a single uniform measure. They were operationalized using individual- and household-level variables such as food insecurity, housing insecurity, utility difficulties, transportation barriers, medication affordability or insurance coverage, caregiver support, and access to healthcare, as well as neighborhood-level composite measures such as the Social Vulnerability Index and Child Opportunity Index [33-35]. The Social Vulnerability Index incorporates domains including socioeconomic status, household composition, housing, and transportation, whereas the Child Opportunity Index captures neighborhood conditions related to education, health and environment, and socioeconomic opportunity [33].

Clinical screening approaches have also measured social needs directly. In a pediatric respiratory clinic, a structured questionnaire assessed medication coverage and out-of-pocket costs, food, utilities and housing insecurity, transportation difficulties, and perceived caregiver support [34]. Other pediatric studies have assessed reported needs in housing, utilities, transportation, and food and examined their associations with healthcare utilization [35]. These heterogeneous approaches indicate that social disadvantage is best interpreted as a multidimensional construct composed of interacting household-, healthcare-, and neighborhood-level factors rather than as a single exposure variable [33-35].

The quality of housing remains relevant to pediatric respiratory health because dampness, inadequate ventilation, pest exposure, overcrowding, and unstable housing may increase respiratory exposures and barriers to disease management [9,28]. Neighborhood-level disadvantage has additionally been examined through geocoded residential data and composite vulnerability or opportunity indices, with greater neighborhood social vulnerability associated with increased pediatric intensive-care use for severe acute respiratory illness [33]. Socioeconomic conditions have also been associated with respiratory outcomes in children with chronic respiratory disease, although the specific pathways and magnitude of these associations vary according to the underlying disorder and population studied [36].

Pediatric respiratory care access also influences disease recognition, continuity of management, and healthcare utilization [9,34,35]. Limited access to care, transportation difficulties, medication costs, and inadequate coverage may create barriers to timely and continuous treatment [34,37]. These factors have been evaluated through measurable outcomes such as positive social-needs screens, social-work referral, emergency or inpatient healthcare utilization, readmission, and hospital length of stay [38,39].

Evidence regarding interventions is more limited than evidence regarding associations. Social screening linked to referral and resource navigation has demonstrated feasibility in pediatric respiratory practice; in one clinic-based study, 69 of 313 screened families were referred to social work and 35 subsequently received an active intervention [34]. However, this study primarily evaluated feasibility and early implementation outcomes rather than demonstrating reductions in asthma exacerbations or improvement in long-term lung function. Therefore, housing assistance, school-based support, medication-access programs, environmental remediation, and similar approaches should not be presented as established effective interventions on the basis of the evidence reviewed here. Instead, they represent potential strategies requiring stronger prospective and intervention-based evaluation for respiratory outcomes. Overall, the available evidence supports associations between social disadvantage, healthcare barriers, and greater pediatric respiratory morbidity, but heterogeneity in how social determinants are measured limits direct comparison across studies. Evidence that screening, housing, access, or other social interventions independently improve asthma control, exacerbation frequency, or long-term lung-function trajectories remains limited and requires prospective evaluation. Table 3 presents key social determinants influencing pediatric respiratory outcomes and care access.

**Table 3:** Social and access-related drivers of pediatric respiratory morbidity. Created by the authors based on information synthesized from previously published studies [30,35,37,39,40]. Therefore, no copyright permission or license agreement was required. ED: emergency department, PM: particulate matter.

Determinant	Main exposure or barrier	Respiratory impact	Potential equity-focused response	References
Poverty	Limited resources, unstable care access	Greater healthcare-use burden and barriers to disease management	Medication-access support and linkage to social resources	[30,40]
Poor housing quality	Dampness, mould, pests, overcrowding	Greater respiratory exposure burden and potential worsening of wheeze or asthma symptoms	Housing assessment and remediation where feasible	[19,30]
Environmental injustice	Traffic, industry, low green space	PM exposure, airway inflammation	Community-level exposure reduction and environmental mitigation	[30,38]
Caregiver/social support needs	Difficulty navigating care, treatment costs, transportation, or limited support	Interrupted care, delayed treatment, and greater healthcare utilization	Structured social-needs screening, referral, and resource navigation	[39,40]
Limited specialist access	Referral, transportation, financial, or coverage barriers	Delayed assessment and disrupted continuity of care	Improved referral pathways and care-access support	[19,39,40]

Long-term health outcomes: Lung-function trajectories, asthma persistence, and adult respiratory risk

Pediatric respiratory morbidity can influence respiratory function across the life course rather than being confined to childhood [37]. Longitudinal cohort evidence demonstrates that lung-function trajectories are established early and may persist into adulthood. In an analysis of six population-based birth cohorts including 8,114 participants in the discovery sample, four trajectories from childhood to early adulthood were identified: normal airflow in 80.8%, persistent airflow obstruction in 15.8%, worsening airflow obstruction in 2.0%, and improving airflow obstruction in 1.5% [37]. Improvement occurred more frequently between childhood and adolescence, whereas worsening airflow obstruction was more prominent between adolescence and early adulthood, demonstrating that respiratory trajectories are dynamic but that a substantial subgroup enters adulthood with persistent impairment [37].

Similar findings have been reported in other longitudinal cohorts. A cohort of 865 participants followed with repeated spirometry from ages 9 to 45 years identified 10 distinct forced expiratory volume in one second (FEV₁) trajectories [38]. The three trajectories resulting in the lowest FEV₁ at age 45 comprised only 19% of the cohort but accounted for 55% of post-bronchodilator airflow obstruction at age 45 [38]. Moreover, trajectories that failed to achieve a normal peak FEV₁ in early adulthood accounted for a substantial proportion of later airflow obstruction, supporting the concept that adult obstructive physiology may originate from impaired lung development rather than exclusively from accelerated decline in adulthood [38].

Repeated spirometry across three population-based birth cohorts identified four childhood FEV₁ trajectories: persistently high, normal, below average, and persistently low [39]. The persistently low trajectory represented approximately 5% of 2,436 participants and was associated with persistent wheezing and asthma throughout follow-up. Recurrent wheeze with severe exacerbations, early allergic sensitization, and tobacco smoke exposure were among the early-life factors associated with this persistently low trajectory [39]. These findings provide direct longitudinal evidence that childhood respiratory and allergic phenotypes can influence the level of lung function attained by early adulthood [39].

Long-term consequences may also extend beyond respiratory disease. In three birth cohorts comprising 6,377 participants, above-average, average, below-average, and persistently low FEV₁/forced vital capacity (FVC) trajectories accounted for 49.5%, 38.3%, 10.6%, and 1.7% of participants, respectively [40]. Wheeze, asthma diagnosis or treatment, and allergic sensitization were consistently associated with diminished trajectories. Lower FEV₁/FVC trajectories were also associated with objective markers of cardiovascular risk in early adulthood, suggesting that impaired childhood lung-function development may be associated with broader adult health risk beyond respiratory impairment alone [40].

Taken together, these longitudinal findings demonstrate that “lung-function tracking” is measurable rather than merely conceptual. Repeated FEV₁, FVC, and FEV₁/FVC measurements across childhood, adolescence, and adulthood identify persistent-low, worsening, improving, and normal trajectories, with adverse childhood trajectories contributing disproportionately to later airflow obstruction [37-40]. The evidence also indicates that childhood asthma, recurrent wheeze, allergic sensitization, and tobacco exposure are associated with poorer trajectories, whereas some children demonstrate partial improvement over time [37-40]. These cohort findings support heterogeneity in respiratory development rather than a deterministic progression from childhood symptoms to adult obstructive disease. Although the consistency of these longitudinal observations strengthens the life-course association, differences in cohort composition, spirometric definitions, trajectory modeling, and follow-up duration limit direct comparisons of effect magnitude across studies. Further harmonized longitudinal studies are needed to improve individual prognostic prediction and determine which modifiable childhood factors most strongly influence adverse adult respiratory trajectories.

Prevention and clinical management

Pediatric respiratory conditions require a layered approach to prevention and clinical management that considers biological susceptibility, environmental exposures, prevention of infection, symptom control, and long-term monitoring [41]. Preventive measures commonly considered during pregnancy and childhood include avoidance of tobacco smoke, improvement of indoor air quality, appropriate nutrition, and prevention of vaccine-preventable respiratory infections [41]. However, the present narrative review did not systematically evaluate the comparative efficacy of each preventive intervention. Accordingly, these measures are presented as clinically relevant preventive approaches rather than interventions demonstrated within this review to alter long-term respiratory trajectories. The available evidence reviewed here does not permit quantification of the independent effect of vaccination or other individual preventive measures on subsequent asthma persistence or long-term lung-function development.

Environmental modification may be appropriate when potentially harmful exposures such as indoor dampness, mould, tobacco or biomass smoke, inadequate ventilation, or traffic-related pollution are identified [11,29-32]. Observational evidence links these exposures with respiratory morbidity, but the intervention evidence reviewed here is insufficient to conclude that removal of any single exposure consistently reduces exacerbations or improves long-term lung-function trajectories [11,29-32]. Therefore, household remediation, school-air-quality measures, and broader pollution-control strategies should be regarded as potential exposure-reduction approaches rather than interventions with established efficacy for altering long-term respiratory outcomes. This distinction is particularly relevant for children with asthma or recurrent wheeze, in whom multiple environmental and clinical determinants may coexist.

The focus of clinical management should be early diagnosis, phenotype recognition, severity assessment, and individualized therapy [42]. Typical elements of asthma care include inhaled corticosteroids, appropriate reliever medication, identification and avoidance of relevant triggers, education regarding inhaler technique, written action plans, and regular reassessment of disease control [42]. Additional controller therapy should be individualized according to age, symptom control, exacerbation history, treatment adherence, and asthma phenotype, with specialist assessment for children whose disease remains poorly controlled despite appropriate standard therapy [42]. Because biologic therapies have age-, phenotype-, and biomarker-specific indications that require evidence from individual clinical trials, specific biologic agents are not recommended within the present review. Antimicrobial stewardship is also relevant because unnecessary antibiotic exposure may contribute to microbiome disruption; however, whether stewardship itself modifies subsequent wheezing or asthma trajectories remains uncertain [24,41].

Long-term follow-up should be individualized according to the underlying respiratory disorder, severity, recurrence of symptoms, and evidence of impaired lung function. In children with asthma or recurrent wheeze, follow-up may include reassessment of symptom control, adherence, inhaler technique, relevant exposures, and pulmonary function where age-appropriate [42]. School-based support, digital monitoring, rehabilitation approaches, and caregiver education may support disease management, but the evidence synthesized in this review does not show that any of these interventions independently improve long-term lung function development. Therefore, their effectiveness should be evaluated in appropriately designed prospective intervention studies. Overall, established management principles support appropriate diagnosis, control of active disease, reduction of avoidable exposures, and longitudinal follow-up, whereas evidence that individual preventive or supportive interventions modify long-term respiratory trajectories remains limited. Future studies should therefore distinguish short-term improvements in symptoms or healthcare utilization from sustained effects on asthma persistence, exacerbation burden, and lung-function development.

Additional clinical context and special-population considerations

Respiratory outcomes in children are influenced not only by disease phenotype and infection history but also by developmental vulnerability, comorbid conditions, socioeconomic circumstances, and the severity of acute respiratory illness. Children with pre-existing respiratory disorders who experience socioeconomic disadvantage may be exposed to a greater cumulative burden through the interaction of adverse living conditions, healthcare-access limitations, and underlying respiratory susceptibility [43]. These factors should be interpreted as contextual modifiers of respiratory risk rather than independent determinants with uniform effects across all pediatric populations.

Host responses to respiratory infection may also vary according to the causative pathogen and the underlying immunological phenotype. Immunoglobulin E-related responses have been reported in association with Mycoplasma pneumoniae infection; however, these findings are pathogen-specific and should not be extrapolated to pediatric asthma or recurrent wheeze more broadly [44]. Similarly, evidence generated during the COVID-19 pandemic demonstrated the importance of age-specific interpretation of respiratory infection in children, particularly because disease manifestations and therapeutic recommendations differ from those in adults [45]. Together, these observations support pathogen- and host-specific interpretation of pediatric respiratory infection rather than assuming that different infectious exposures share equivalent mechanisms or long-term consequences.

Certain pediatric populations have distinct long-term respiratory vulnerabilities. Children born prematurely who develop bronchopulmonary dysplasia may experience persistent abnormalities in pulmonary structure and function beyond infancy, supporting the need for continued respiratory surveillance during childhood [46]. In addition, population-level variations in bronchiolitis-related hospitalization indicate that the burden of early-life respiratory disease is influenced by demographic, epidemiological, and healthcare-system factors [47].

Children with neurological and developmental disorders constitute another clinically important high-risk group. In cerebral palsy, respiratory morbidity may be related to impaired airway clearance, aspiration, reduced mobility, and recurrent infection. Preventive respiratory-care strategies have been evaluated in this population, although the available evidence remains limited and should not be generalized to otherwise healthy children [48]. Likewise, influenza contributes substantially to pediatric respiratory disease burden, supporting the clinical relevance of preventing vaccine-preventable respiratory infections while recognizing that disease-burden evidence alone does not establish an effect of vaccination on subsequent asthma persistence or long-term lung-function trajectories [49].

The relevance of childhood respiratory impairment may extend into adult life. Persistent airflow limitation in adulthood is associated with chronic obstructive respiratory disease and reduced functional respiratory reserve [50]. However, childhood respiratory morbidity should not be interpreted as a deterministic precursor of adult chronic obstructive pulmonary disease. Rather, failure to attain optimal peak lung function during childhood and adolescence may represent one of several pathways contributing to reduced respiratory reserve and greater susceptibility to chronic respiratory disease later in life. Overall, the evidence from these special populations broadens the life-course framework of pediatric respiratory disease but remains heterogeneous in design, population, and outcome assessment. These studies therefore provide contextual and supportive evidence rather than a basis for generalizing specific causal pathways or intervention effects across all children. Collectively, these findings support a life-course framework in which developmental vulnerability, acute respiratory insults, comorbid conditions, environmental exposures, and healthcare context interact to shape long-term respiratory outcomes.

Limitations and future recommendations

This review is limited by variability in study designs, exposure assessment methods, diagnostic definitions, geographic settings, and follow-up durations across the included literature because the evidence was synthesized narratively rather than through a quantitative meta-analysis, the relative magnitude, statistical significance, or independent contribution of each clinical, immunological, environmental, and social determinant could not be objectively ranked. Many included studies were observational and used heterogeneous measures of exposure and outcome, precluding calculation of pooled effect estimates or direct comparison of risk strength across determinants. Accordingly, terms such as “important” or “major” determinants in this review should be interpreted as reflecting the consistency and clinical relevance of reported associations rather than a quantitatively established hierarchy of risk. Causal interpretation is also limited because many studies are potentially confounded by socioeconomic conditions, housing characteristics, healthcare access, comorbid atopy, and regional patterns of pollution. Evidence remains particularly uncertain regarding causal microbiome signatures, the magnitude of climate-related respiratory effects, and the persistence of post-viral respiratory consequences across childhood and into adulthood.

The purpose of this review was not to identify previously unreported risk factors or establish new causal associations. Rather, it was to integrate established and emerging determinants across clinical, immunological, infectious, environmental, and social domains and examine how they may jointly influence long-term respiratory trajectories. The synthesis did not identify a sufficiently consistent protective or negative association that could be considered a new determinant. However, it highlighted areas in which the direction or magnitude of associations remains uncertain, particularly microbiome-related factors, climate-related exposures, and the long-term consequences of early viral infections. These uncertainties should be interpreted as evidence gaps rather than as evidence of the absence of an association.

Future studies should focus on longitudinal pediatric cohorts that are carefully phenotyped for respiratory diseases and incorporate standardized, objective exposure measures, repeated spirometry, validated immunological and biomarker assessments, and multivariable analyses capable of estimating the independent contribution of individual determinants. Where feasible, repeated measurements across infancy, childhood, adolescence, and early adulthood would help distinguish transient associations from persistent developmental trajectories. Where sufficient homogeneous data become available, future systematic reviews should use meta-analytic methods to generate pooled effect estimates and permit objective comparison of risk magnitude. Further work is also needed on exposome-based models, biomarker-guided risk stratification, microbiome-immune interactions, and health-equity-focused interventions. Intervention studies should specifically test whether modifying environmental exposures, inflammatory pathways, social barriers, or healthcare-related determinants changes clinically meaningful outcomes such as exacerbation frequency, asthma persistence, or lung-function trajectories, rather than inferring benefit from observational associations alone.

## Conclusions

Pediatric respiratory diseases reflect a complex interplay of clinical vulnerability, immune maturation, environmental exposures, infection history, and social determinants. Early-life factors such as prematurity, impaired lung development, recurrent viral lower respiratory tract infections, and atopic susceptibility may contribute to persistent wheeze, asthma, and suboptimal lung function development. Type 2 inflammation, epithelial barrier dysfunction, impaired antiviral responses, eosinophilic activity, and microbiome disruption are involved in mechanisms associated with recurrent wheeze, asthma persistence, and airway hyperresponsiveness. Environmental exposures, including biomass smoke, indoor dampness, allergens, tobacco smoke, air pollution, and climate-related hazards, may further increase respiratory morbidity, although their effects vary according to exposure intensity, timing, and underlying susceptibility. Social disadvantage may influence both exposure burden and access to healthcare, contributing to inequities in disease recognition, continuity of care, and respiratory outcomes. Longitudinal evidence indicates that adverse childhood lung-function trajectories can persist into adulthood, while some children demonstrate partial improvement, highlighting substantial heterogeneity in respiratory development. The available evidence is stronger for identifying associations and long-term risk trajectories than for demonstrating that individual preventive or therapeutic interventions alter those trajectories. Clinical care should therefore emphasize appropriate diagnosis, phenotype- and severity-based management, reduction of avoidable exposures, age-appropriate pulmonary-function monitoring, and equitable access to follow-up, while the long-term effectiveness of specific preventive strategies requires further prospective evaluation.
